# The Molecular Basis of Multiple Morphological Abnormalities of Sperm Flagella and Its Impact on Clinical Practice

**DOI:** 10.3390/genes15101315

**Published:** 2024-10-13

**Authors:** Yujie Zhou, Songyan Yu, Wenyong Zhang

**Affiliations:** 1 School of Medicine, Southern University of Science and Technology, Shenzhen 518055, China; 12110522@mail.sustech.edu.cn (Y.Z.); 12112023@mail.sustech.edu.cn (S.Y.); 2 Key University Laboratory of Metabolism and Health of Guangdong, Southern University of Science and Technology, Shenzhen 518055, China

**Keywords:** MMAF, rare disease, infertility, ICSI, molecular mechanism, sperm motility

## Abstract

Multiple morphological abnormalities of the sperm flagella (MMAF) is a specific form of severe flagellar or ciliary deficiency syndrome. MMAF is characterized by primary infertility with abnormal morphology in the flagella of spermatozoa, presenting with short, absent, bent, coiled, and irregular flagella. As a rare disease first named in 2014, studies in recent years have shed light on the molecular defects of MMAF that comprise the structure and biological function of the sperm flagella. Understanding the molecular genetics of MMAF may provide opportunities for the development of diagnostic and therapeutic strategies for this rare disease. This review aims to summarize current studies regarding the molecular pathogenesis of MMAF and describe strategies of genetic counseling, clinical diagnosis, and therapy for MMAF.

## 1. Introduction

Infertility is a serious health issue affecting approximately 10–20% of couples globally, with male factors contributing to nearly half of these cases [1]. Among the male factors, multiple morphological abnormalities of the sperm flagella (MMAF) stands out as a significant cause of male infertility, leading to asthenozoospermia. MMAF is characterized by multiple morphological defects of the sperm flagella, including short, coiled, absent, and irregularly shaped flagella, which severely compromise sperm motility [2]. The structural integrity of the sperm flagellum is crucial for its motility and overall fertilization potential, making these abnormalities a key focus in the study of male infertility.

Since the identification of the first MMAF-associated gene, *DNAH1*, research has rapidly expanded, uncovering more than forty genes linked to this syndrome through various mechanisms [3]. These genes include those encoding flagellar structural proteins(DNAH2 [4], DNAH6 [5], and DNAH17 [6]), centrosomal proteins (DZIP1 [7] and CEP135 [8]), and intra-flagellar transport related proteins (TTC21A [9] and CCDC34 [10]). Despite these advances, the pathogenesis of MMAF remains largely unknown, and many patients still lack a definitive causal diagnosis. This highlights the complexity of MMAF and the need for continued research to fully elucidate the underlying genetic and molecular mechanisms. Recent years have witnessed significant progress, with the discovery of new mutations and further insights into previously obscure protein functions. For instance, in mouse models, *CCDC189* has emerged as a new candidate gene for MMAF, localizing to the radial spoke of the first peripheral microtubule doublet in the sperm axoneme [11]. 

Intracytoplasmic sperm injection (ICSI) remains the most widely applied assisted reproductive technology (ART) and is effective in treating the majority of MMAF patients [12]. While many MMAF patients achieve successful outcomes with ICSI, there are still cases of treatment failure. Ongoing research is shedding light on the relationship between the efficacy of ICSI treatment and the proteins affected by genetic mutations, providing a deeper understanding of the factors influencing ICSI success in MMAF patients.

In this review, we aim to summarize the latest findings on the genetic causes of the MMAF phenotype, with a particular focus on the underlying molecular mechanisms. We will also discuss insights into the diagnosis of MMAF and the implications for ICSI treatment, offering a comprehensive overview of the current state of knowledge in this field.

## 2. Multiple Morphological Abnormalities of the Sperm Flagella (MMAF)

### 2.1. Normal Sperm Flagella Structure

**Figure 1:**
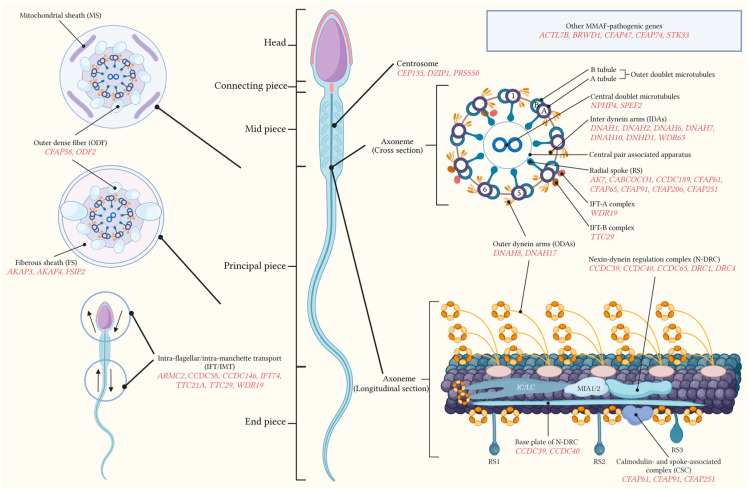
Sperm flagellar structure and known genetic mutations associated with MMAF. Pathogenic genes are grouped according to their structural locations or biological processes. Other pathogenic genes associated with MMAF are placed in the blue box located in the upper right corner.

As the site of defects associated with the etiology of MMAF, the structure of sperm flagella can be divided into four parts—the connecting piece connected to the head of the sperm, the mid piece, the principal piece, and the end piece, as shown in Figure 1. The whole length of the sperm flagellum is covered by the axoneme, which is the central supporting structure of the sperm tail. The axoneme is formed by the centrosome attaching to the basal plate. Several gene mutations are associated with the centrosome such as *CEP135*, *DZIP1,* and *PRSS50*. Additionally, the axoneme is surrounded by structures called peri-axonemal structures, except for the end piece. The ultrastructure of the axoneme shows nine outer doublet microtubules, and one central microtubule pair (the “9 + 2” structure) occupies the core position. The mutated genes associated with the central pair include *NPHP4* and *SPEF2*. The cross-section of the axoneme is mainly composed of the radial spokes (RSs), inner dynein arms (IDAs), and outer dynein arms (ODAs). A large number of mutations are related to these structures, for instance, RS (*AK7*, *CCDC189*, *CFAP65*, etc.), IDA (*CFAP43*, *CFAP44*, *DNAH1*, etc.) and ODA (*CFAP70*, *DNAH8*, *DNAH17*, etc.). The longitudinal section of the axoneme mainly consists of the nexin–dynein regulation complex (N-DRC) and the calmodulin- and spoke-associated complex (CSC). The N-DRC can be impacted by mutations in genes such as *CCDC39*, *CCDC40*, *DRC1*, etc. Meanwhile, the CSC-associated genes implicated in MMAF include *CFAP61*, *CFAP91,* and *CFAP251*. The peri-axonemal structures are composed of different substructures in different positions of the flagellum. As the protective element, the outer dense fibers (ODFs) cover the axoneme with nine columns extending from the connecting piece to the principal piece. Mutations in *CFAP58* and *ODF2* can disrupt the normal ODFs. In the midpiece, the mitochondrial sheath (MS) is present beneath the plasma membrane spiral around the ODF and the structures within it. In the principal piece, the MS is gradually replaced by the fibrous sheath (FS) which features mutations in *AKAP3*, *AKAP4,* and *FSIP2*. Flagellar assembly processes including the intra-flagellar transport (IFT) and the intra-manchette transport (IMT) are also significant components of the sperm tail. The associated genes include *ARMC2*, *CCDC38*, *IFT74* and *WDR19*. The existence of other MMAF-associated genes such as *ACTL7B* and *BRWD1* suggests that more precise gene–structure or gene–function relationships remain to be discovered.

### 2.2. Concept of MMAF

Although the concept of “multiple morphological anomalies of the flagella (MMAF)” was only first proposed in 2014, the disease had been recognized and described as “tail defects in the spermatozoa”, “tail stump spermatozoa”, “short tail defect in human spermatozoa”, or “male infertility due to flagellar anomaly” over the past 40 years [3,13,14,15,16]. At first, this type of disease was recognized as a phenotype of primary ciliary syndrome due to the conservative axonemal structure of cilia and sperm flagella [17]. However, this type of asthenozoospermia had been found in infertile men with no other symptoms and had been linked to widespread abnormalities in the morphology of the sperm flagella [18,19]. Then, a more accurate description of this disease, MMAF, appeared at the beginning of the exploration of genetic pathogenicity. It refers to an extreme condition in which sperm motility is nearly completely lost [3]. At present, the definition of MMAF mainly focuses on the abnormal morphological characteristics of sperm flagella, including short, absent, bent, coiled, and irregular flagella. This mosaic of abnormal morphology of the sperm tail can be easily identified by routine semen analysis under a light microscope based on the procedural evaluative criteria in the Fifth and Sixth World Health Organization (WHO) Laboratory Manuals for the Examination and Processing of Human Semen [20,21]. The initial diagnosis of MMAF requires the detection of over 5% sperm with at least four kinds of the flagellar morphological abnormalities mentioned above [3]. These defects have been related to disorders in axonemal and peri-axonemal structures at the ultra-structural level [22]. Furthermore, genetic analysis has greatly improved the diagnostic accuracy of MMAF. A practical diagnostic protocol for MMAF has emerged including the identification of an abnormal sperm motility percentage, ultrastructure observation, and genetic analysis [23]. 

### 2.3. Differential Diagnosis of MMAF

The concept of MMAF should be distinguished with other similar diseases belonging to the realm of asthenozoospermia. Asthenozoospermia mainly includes MMAF and primary ciliary dyskinesia (PCD), which are both the results of disorders in the ultrastructural flagellar or ciliary structures. However, not all patients with PCD present as infertile or have ultrastructural defects in the axoneme. PCD is mainly characterized by deficiency in mucociliary clearance and chronic airway disease. In addition, MMAF is a primary morphological change in the flagella and is relatively restricted to the flagella. Primary MS defects in MMAF can lead to specific phenomena such as the lack of an annulus between the mid and principal pieces [24]. Also, differences between PCD and MMAF may depend on the nuances in the axonemal components and structures. For instance, the loss of ODAs caused by the *DNAH17* mutation is sperm-specific [25]. Moreover, other causes of male infertility should be considered in the clinical diagnosis. For example, environmental pollution has been revealed to be associated with morphological alteration of the sperm, according to Perrone et al. [26]. However, this kind of morphological abnormality mainly manifests in the sperm head, which can be distinguished from MMAF.

## 3. Molecular Genetics of MMAF

More than 40 associated genes have been identified in association with MMAF since 2014. The structural components and their associated MMAF pathogenic genes are shown in the following figure based on the current knowledge (Figure 1).

### 3.1. Axonemal-Associated Pathogenic Genes

The typical structure of the axoneme is the “9 + 2” structure based on the microtubules and other structures attached to the outer doublet microtubules, including the ODA, the IDA, the N-DRC, and the RS. As a supramolecular machinery, the axoneme depends on the above structures and their organized interactions to maintain the stability of itself and the whole sperm flagellum. The axoneme can cover the full length of the flagellum, especially including the end piece. Due to the significance of the axoneme in the function and composition of flagella, any defects in the axoneme components would lead to morphological disorder of the sperm flagellum. The axonemal-associated MMAF genes are summarized in Table 1. 

#### 3.1.1. Gene Mutations Related to the Outer Dynein Arm (ODA)

The dynein arms are microtubule-dependent ATPases anchored to the A tubules of outer doublet microtubules. Three motor subunits, namely the heavy chain, the intermediate chain, and the light chain, comprise the dynein arms to motivate the motility of sperm flagella. The outer dynein arm (ODA) can promote the frequency of the flagella oscillations, regulate the propagation of asymmetric waveforms, and further produce power for the sperm. 

In the last five years, studies of ODA-related genetic mutations have emerged in the etiological exploration of MMAF. Current known pathogenic genes associated with ODA include the dynein heavy chain 8 gene (*DNAH8*), *DNAH17,* and cilia- and flagella-associated protein 70 gene (*CFAP70*).

CFAP70 was first reported in 2018 as a novel regulatory protein of ODAs, especially regulating the frequency of flagella beating in mice. Meanwhile, it was discovered that the CFAP70 homolog in *Chlamydomonas*, FAP70, resides at the base of ODAs [77]. As research continued, Beurois et al. showed that two MMAF patients carried homozygous mutations in the *CFAP70*, c.1723-1G>T and c.178T>A. Also, one patient presented ODA defects described in a case report [27]. The regulatory impact of *CFAP70* on the expression of proteins such as QRICH2 and TTL5 was suggested [28]. Then, studies revealed that CFAP70 protein could bind to calmodulin- and spoke-associated complex (CSC) components such as CFAP61, CFAP91, and CFAP251 to modulate dynein activity [27,28]. In 2023, Chen et al. provided evidence that CFAP70 can interact with ODA components dynein axonemal intermediate chain 1 (DNAI1) and DNAI2 and become involved in the transport of flagella constitutions via a-kinase anchoring protein 3 (AKAP3) in a *Cfap70*-deficient mouse model [29].

Both DNAH8 and DNAH17 are significant components of ODA heavy chains between which the interaction exists. *DNAH8* bi-allelic and frameshift variants have been found in MMAF patients with disintegrated or disappeared ODAs [31]. In addition, the same study showed that *Dnah8*-KO male mice displayed classical MMAF phenotypes [31]. With *DNAH8* frameshift variants discovered in MMAF patients, DNAH8 is thought to play a role in the spermatogenesis and assembly of the axoneme since *DNAH8* gene mutations led to serious disorders of the central microtubule pair in MMAF patients [30,32]. Similarly, several studies have reported a large number of *DNAH17* gene mutations related to the dynein heavy0chain domain, ATPase domain, and microtubule-binding domain of the DNAH17 protein in MMAF patients all over the world [6,33,34,35,36,37]. DNAH17 plays a specific role in sperm flagella formation due to the lack of ODAs in these MMAF patients [33,34]. Interestingly, a study in 2024 showed that human tetratricopeptide repeat domain 12 (TTC12), a protein involved in the assembly of dynein arm complexes, can impact the expression of DNAH17 [78].

#### 3.1.2. Gene Mutations Related to the Inner Dynein Arm (IDA)

The inner dynein arm (IDA) plays an important role in the formation of the flagellar flexural beat and the propagation of waveforms and amplitudes. As the most classic MMAF pathogenic genes, IDA-associated genes in MMAF have expanded from *DNAH1*, *DNAH2*, *DNAH6*, *DNAH7*, *DNAH10*, *CFAP43*, *CFAP44*, dynein heavy-chain domain 1 gene (*DNHD1*), and WD repeat-domain 63 protein gene (*WDR63*) since the first MMAF gene was reported in 2014.

Frameshift, nonsense, and splice-site mutations in *CFAP43* and *CFAP44* have been reported in MMAF patients [38,39,40,41,42,79]. Patients with *CFAP43* and *CFAP44* defects could account for 30.8% of all MMAF patients [41]. Although FS hyperplasia and the disorganization of microtubules have been discovered in patients and KO mice with *CFAP43* and *CFAP44* mutations, *CFAP43* mutations seem to be related to more severe phenotypes [39]. CFAP43 and CFAP44 proteins are presumed to be located next to the outer doublet microtubules 5–6 bridge and approach to the IDAs [80,81]. On the one hand, CFAP43 and CFAP44 proteins may assist the outer doublet microtubules 5–6 bridge in stabilizing the axonemal and peri-axonemal structures [39]. On the other hand, CFAP43 and CFAP44 proteins may interact with the IDAs to regulate the motility of the flagella. This is proposed because it has been confirmed that the orthologous Fap43p and Fap44p proteins in *Tetrahymena* connect the motor domain of IDA I1 to the outer microtubule doublet [81]. In 2021, Yu et al. suggested that Cfap43 protein can participate in the intra-manchette transport (IMT) associated with flagellar assembly in mice, which broadens the possible functions of CFAP43 in MMAF [43].

*DNAH1* is the first discovered and globally accepted MMAF-pathological gene [3]. *DNAH1* mutations have been considered a main cause of MMAF, with the estimated rate of *DNAH1* mutations in MMAF at 24.6% [2]. Although a large number of *DNAH1* mutations in MMAF patients have been reported, the novel mutations and mutational landscape of *DNAH1* in certain regions have yielded new findings recently [44,45,46,47,48,49]. Dacheux et al. reported that a novel axonemal protein, zinc finger MYND-type containing 12 (ZMYND12), can impact the stability of the axoneme through the interaction with DNAH1 in RS3 base-docked IDAs [82]. *DNAH2* mutations in MMAF patients were first reported in 2019 [4]. In this study, five *DNAH2* mutations (c.9298C>T, c.5770C>T, c.11500C>T, c.6960C>A, and c.11503T>C) were discovered, including nonsense and missense mutations [4]. Interestingly, the c.9298C>T variant is located at the microtubule-binding site of *DNAH2* [4]. Then, Gao et al. found six novel bi-allelic variants of *DNAH2* in a cohort of MMAF patients. Significantly, these *DNAH2* variants can block the ATPase activity of DNAH2 protein by altering the three-dimensional structure of this protein [50]. In addition, the MMAF phenotypes could also be observed in *Dnah2* knockout mice [51]. The expression of WDR78, which is a component of the dynein light chain, can interact with DNAH2 to impact the flagella beating [83]. *DNAH6* mutations were first reported in MMAF patients in 2019 [52]. Although the detailed molecular mechanism of *DNAH6* mutations in causing MMAF is unclear, it is speculated that these identified heterozygous mutations in *DNAH6* can lead to MMAF by disrupting the normal structure of DNAH6 protein as an ATPase and microtubule-binding protein [52]. Interestingly, the level of DNAH1 protein was also reduced in these patients [52]. Shao et al. observed coiled sperm flagella with the affected RS and the absence of the central pair in patients with the novel *DNAH6* variants [5]. *DNAH7*, another member of the gene family controlling the expression of the dynein heavy-chain protein, was shown to be related to MMAF. The study described how *DNAH7* nonsense variants were present in an infertile man whose sperm showed short or coiled flagella and severe loss of IDAs [53]. The authors speculated that DNAH7 could be involved in the formation of the MS-regulatory complex with male germ cells Rab GTPase-activating protein (TBC1D21) and translocase of outer mitochondrial membrane 20 (TOMM20) [53]. Wu et al. suggested that DNALI1 may regulate the cytoplasmic dynein complex, and that *DNALI1* deficiency could result in abnormalities of the flagellar FS and IDAs [84]. *DNAH10* is also a novel MMAF-pathogenetic gene whose bi-allelic variants have been identified in MMAF patients [54,55]. Knockout of *Dnah10* in mice leads to the MMAF phenotype [54]. In addition, some *DNAH10* mutations seem to be the secondary causes of MMAF accompanied by defects in DNAH1, DNAH2, and DNALI1 proteins [55]. WDR63 is a vital component of the IDA intermediate chains. Lu et al. reported bi-allelic variants of *WDR63* in infertile men with MMAF symptoms, and a KO mice model was created to confirm its functional role [57]. Another gene coding one of the dynein heavy-chain domains, *DNHD1*, has been implicated in MMAF according to Martinez et al. The authors discovered that recurrent or atypical MMAF phenotypes such as defects in the central pair or MS can be a result of missense and stop-gain mutations in *DNHD1* [56].

#### 3.1.3. Gene Mutations Related to the Nexin-Dynein Regulation Complex (N-DRC)

The nexin–dynein regulation complex (N-DRC) is a Y-shaped complex bridging the adjacent outer doublet microtubules from the base plate on the A tubule to the linker region on the neighboring B tubule. As a result, the N-DRC limits sliding between adjacent microtubules in order to transform the sliding motion produced by dynein to flagellar beating [85]. In recent years, the MMAF-pathogenetic genes related to the N-DRC mainly include dynein regulatory complex subunit 1 protein gene (*DRC1*), *DRC4*, coiled-coil domain containing 39 gene (*CCDC39*), and *CCDC40*.

CCDC39 and CCDC40 proteins are the base plate components of N-DRC. New variants in *CCDC39* and *CCDC40* have been identified to be associated with both PCD and MMAF [58,59]. Aprea et al. verified that MMAF ultrastructural phenotypes such as unassembled axonemal and peri-axonemal components were present in PCD patients with several *CCDC39* and *CCDC40* variants [60]. This study demonstrated the interaction between CCDC39 and CCDC40 in the sperm flagella, which provided a new direction for future research [60]. Also, another N-DRC-associated PCD gene *CCDC65* has been verified to be related to MMAF. In MMAF patients, *CCDC65* variants appeared simultaneously with the absence of other N-DRC, ODA, and IDA components including DRC4, DNAI1, and DNALI1 [61]. DRC1 and DRC4, also located on the base plate and serving as the segments of the N-DRC core structure, are both the pathogenetic factors of MMAF. Different kinds of *DRC1* mutations have been observed in MMAF patients and patients with both PCD and MMAF [62,63]. In addition, *DRC1* defects could trigger MMAF phenotypes such as disordered microtubules in *Drc1^−/−^*, *Drc1*^R554X/R554X^, and *Drc1*^W244X/W244X^ mice [63]. DRC4, also known as growth arrest-specific 8 protein (GAS8), was shown to be a possible pathogenic factor of MMAF since a novel splice donor mutation of *GAS8* was observed in MMAF patients [64].

#### 3.1.4. Gene Mutations Related to the Radial Spoke (RS)

The radial spoke (RS) is a T-shaped protein complex bridging the outer and central doublet microtubules. The radial spokes in the flagellum can be divided into three types, including RS1 next to the IDA, RS2 attached to the N-DRC, and RS3 with a different asymmetrical shape [86]. The calmodulin- and radial-spoke-associated complex (CSC) is a structure connecting the RS3 to the RS2 and N-DRC, which is also essential to the flagellar motility [87]. So far, the MMAF-pathogenetic genes related to RS mainly include CSC component genes such as *CFAP61*, *CFAP91* (*MAATS1*), and *CFAP251* (*WDR66*). Recently, it has been reported that MMAF can also be caused by other proteins related to RS in terms of location and function, including adenylate kinase 7 (AK7), CCDC189, and CFAP206.

Among the three components of the CSC, only CFAP61 has been verified to be associated with MMAF in both MMAF patients and mice models [67,68,69,70]. Most of the known *CFAP61* mutations lead to the loss of key structures in CFAP61 protein and further destabilize the RS3. Interestingly, Ma et al. implied the key role of CFAP61 in the CSC since *CFAP61* variants cannot express any CFAP251 protein [68]. *CFAP91* bi-allelic variants in an MMAF patient cohort were reported in 2020. Martinez et al. also confirmed the interaction between CFAP91 and CFAP251 in their article [72]. Finally, a splicing mutation of CFAP251 was discovered in a patient with MMAF-like symptoms [74].

AK7 is thought to participate in regulating the protein kinase A located at the RS. Recent studies have detected nonsense and missense mutations of *AK7* in MMAF patients displaying mitochondrial vacuolization and axonemal disorganization [65,66]. However, the detailed role of *AK7* deficiency remains to be explored. CCDC189 is a protein attached to the RS corresponding to the first pair of outer doublet microtubules. Wang et al. revealed that *Ccdc189*^−/−^mice presented MMAF-like phenotypes such as the coiled FS and impaired MS [11]. Furthermore, these authors found that another novel flagellar protein, called ciliary-associated calcium-binding coiled-coil protein 1 (CABCOCO1), can interact with both CCDC189 and radial spoke head 1 homolog protein (RSPH1) [11]. CFAP206 is a microtubule-docking adapter for the RS and IDA. A novel frameshift mutation in *CFAP206* (c.1430dupA) accompanied by the absence of CFAP251 (WDR66) and RSPH1 was discovered in MMAF patients [73]. This suggests the close relationship between CFAP206 and CSC in regulating the assembly and stability of flagella. Bent and coiled flagella appeared in the *Cfap206*^−/−^ mice [73]. 

CFAP65 is an MMAF-associated protein expressed in the midpiece of the sperm flagella. However, the detailed location and biological function of CFAP65 are still unknown. Significantly, Wang et al. demonstrated that RSPH1 decreased in *Cfap65*^−/−^ mice and that CFAP65 can interact with RSPH1 [71]. This finding suggests that CFAP65 may be a component of the CSC.

#### 3.1.5. Gene Mutations Related to the Central Doublet Microtubules

The central doublet microtubules or central pair are composed of C1 and C2 connected by a bridge-like structure. They play a regulatory role in the sliding of the microtubule in order to produce normal waveforms. Recently, only two genes related to central doublet microtubules, nephrocystin-4 gene (*NPHP4*) and sperm flagellar 2 gene (*SPEF2*), have been implicated in MMAF.

NPHP4 is a structural protein in the cilia. However, a missense mutation (c.1490C>G) in *NPHP4* appeared in MMAF patients with massive loss of the central pair, which implies the relationship between NPHP4 deficiency and the central pair in the pathogenesis of MMAF [75]. SPEF2 is a component of the central-pair-associated apparatus C1b. SPEF2 defects have been identified to cause severe MMAF-like phenotypes such as the scattered MS, ODFs, and FS in patients and *Spef2*^−/−^ mice, according to Tu et al. [76].

### 3.2. Peri-Axoneme-Associated Pathogenic Genes

Peri-axonemal structures, including the outer dense fibers (ODFs) and fibrous sheath (FS) in the midpiece, as well as the ODFs and mitochondrial sheath (MS) in the principal piece, provide additional flagellar stiffness and generate the functional effect of increasing bend wavelength, as well as contribute to the preservation of flagellar integrity [88]. The gene mutations associated with the peri-axonemal structures are listed in Table 2.

ODF2, a critical component of the sperm tail, plays a key role in the assembly of ODFs [98]. Previously, a case of MMAF caused by an *ODF2* mutation was reported, which is the first documented instance of an *ODF2* mutation identified in human sperm [89]. The patient’s sperm exhibited predominantly malformed tails and varying degrees of loss of the outer dense fibers, yet the patient successfully fathered a healthy child through ART [89]. Notably, this case was attributed to a maternally inherited single heterozygous mutation, presenting a dosage-dependent phenotype and revealing an unconventional inheritance pattern [89]. Two cohort studies have reported axonemal and peri-axonemal structural abnormalities in patients with bi-allelic mutations in *CFAP58*. The deficiency of CFAP58 likely disrupts the transport of ODFs, leading to the disorganization of ODF structures [90,91].

Several genes have been implicated in the formation of the fibrous sheath (FS), including *FSIP2*, *AKAP3*, and *AKAP4*, although the precise mechanisms by which deficiencies in these gene-encoded proteins lead to MMAF remain inadequately understood. Fibrous sheath-interacting protein 2 (FSIP2), encoded by *FSIP2*, is involved in the assembly of the FS and constitutes a significant component of the peri-axonemal structures alongside other FS proteins [23]. Multiple mutations in *FSIP2* have been reported to be associated with the MMAF phenotype [92,94,95,99,100]. Further research has revealed that FSIP2 also plays a role in acrosome formation and exhibits dosage-dependent effects [93,101]. A recent study suggests that, in addition to its role in FS assembly, FSIP2 may participate in the assembly of the flagellum and MS as part of the intra-flagellar transport complex [102]. AKAP3 and AKAP4 are structural proteins necessary for anchoring protein kinase A (PKA) to the FS, and they can interact with FSIP2 [23]. AKAP3 contains two domains: the N-terminal RII binding domain, which binds the regulatory subunit of PKA, and the C-terminal domain, which interacts with AKAP4 [103,104,105]. Although homozygous mutations in *AKAP3* have been identified in two MMAF patients [96], a study based on structural modeling and in silico analysis of single-nucleotide polymorphisms (SNPs) found that several variants, including the c.1499C>T variant identified in MMAF patients, did not lead to changes in the secondary structure of the AKAP3 protein [105]. This is consistent with the findings of Turner et al. [106]. Zhang et al. investigated MMAF patients from three different families carrying hemizygous *AKAP4* variants and found dysplasia of the FS. Further research indicated that normal expression of AKAP4 is essential for maintaining the proper expression of glutamine-rich protein 2 (QRICH2). The loss-of-function missense variant of *AKAP4* affects the interaction with QRICH2, leading to FS dysplasia [97].

### 3.3. Centrosome-Associated Pathogenic Genes

The centrosome plays a critical role in sperm formation, fertilization, and the completion of replication and division [107]. The MMAF-pathogenic genes associated with the centrosome are displayed in Table 3.

*CEP135*, which is associated with centrosome biogenesis and the assembly of centrioles (CP), has been reported in patients with MMAF [8]. In this study, researchers discovered that CEP135 protein accumulates as aggregates in the sperm of affected individuals, suggesting that induction of large filamentous aggregates may disrupt centrosome biogenesis, ultimately leading to the MMAF phenotype [8]. The zinc finger-and coiled-coil-containing protein encoded by *DZIP1* has been identified as an essential protein required for sperm flagella and centrosome formation, with a homozygous mutation in this gene causing severe MMAF [7]. Due to the absence of DZIP1, over 90% of the sperm in affected patients lack flagella, a phenotype also observed in *Dzip1*-knockout male mice [7]. CEP78, encoded by *CEP78*, is a centriole wall protein localized to mature centrioles and involved in the regulation of centrosome duplication [110]. The typical MMAF phenotype has been observed in both affected patients and *Cep78*-knockout mice [111]. CEP78 has also been reported to interact with IFT20 and TTC21A, and their interaction and stability are crucial for regulating the length of centrioles and cilia [109]. Another study highlighted the complexity of the protein networks and molecular mechanisms governing sperm flagella assembly and organization. Specifically, a PRSS50/NF-κB/LRWD1 pathway exists, where the absence of PRSS50 leads to the downregulation of LRWD1, resulting in incorrect centrosome formation. Meanwhile, the total H3 levels, along with modified H3K9me1, H3K9me3, and H2AXs139P, decrease. The above dysregulation may be associated with increased AKAP4, impacting spermatogenesis [108]. 

### 3.4. Pathogenic Genes Related to the Flagellar Assembly

Intra-flagellar transport (IFT) is a bidirectional process along axonemal microtubules (MTs) that transports multisubunit protein complexes and plays a crucial role in the loading and function of cilia and flagella [112]. Intra-manchette transport (IMT) is another protein transport system involved in flagellar assembly and nuclear remodeling [113]. Numerous articles provide evidence that abnormalities in IFT and IMT-related genes lead to MMAF (Table 4).

TTC29 is part of the IFT-B complex [122] and may also be involved in flagellar beating [123]. Decreased sperm motility and flagellar ultrastructural abnormalities have been observed in males with *TTC29* mutations and in *Ttc29*-mutant mouse models, confirming that *TTC29* mutations cause male infertility due to MMAF [124]. A more recent study reported novel bi-allelic *TTC29* mutations and found that affected patients not only exhibited the typical MMAF phenotype but also had abnormalities in sperm heads and acrosomes [114]. *CCDC38* is a testis-specific gene localized to the manchette and sperm tail during spermatogenesis [117,125]. Studies have shown that CCDC38 interacts with CCDC42, IFT88, and CFAP53, regulating cargo transport through IMT and IFT during flagellum biogenesis [117]. Another highly expressed testis protein, CCDC46, was discovered to interact with CCDC38, CCDC42, and the IFT complex, affecting ODF transport [118]. ARMC2 is essential for sperm flagella structure and assembly. To date, *ARMC2* mutations have been reported in four studies involving MMAF patients, with strong evidence supporting the notion that *ARMC2* mutations are pathogenic for MMAF [119,120,121,126]. Since flagella remain present in sperm from patients with *ARMC2* mutations, it is hypothesized that ARMC2 might specifically participate in the loading or stabilization of the central pair complex (CPC). However, only Wang et al. have preliminarily explored the interactions of ARMC2 with multiple proteins, and more detailed mechanisms have yet to be studied [120]. Additionally, *WDR19* [115], *TTC21A* [9], *IFT74* [116], and *CCDC34* [10] have been reported to play roles in flagellar assembly, and mutations in these genes can cause MMAF.

### 3.5. Other Pathogenic Genes

We will briefly introduce several other MMAF-related genes that cannot be categorized with the other classes of genes discussed above. These genes are listed in Table 5. 

A report published in 2021 on *CFAP47* mutations and MMAF suggested that MMAF can be caused by hemizygous mutations in an X-linked gene [127]. The in silico tool STRING predicted that CFAP47 might have potential interactions with CFAP65; however, the molecular mechanism by which *CFAP47* variants cause the MMAF phenotype in sperm has not been elucidated [127]. A subsequent study showed that CFAP47 regulates sperm morphological development by modulating the expression of CFAP65, CFAP69, and SEPTIN4 [128]. CFAP69 is a key protein involved in sperm flagella formation [134]. SEPTIN4 is important to the formation of the sperm annulus [135]. This finding explains not only the abnormal sperm head morphology and annulus defects in addition to the MMAF phenotype but also suggests that CFAP47 may play a role in multiple processes during spermatogenesis [128]. Another study discovered that WDR87, a protein localized to the middle piece of the sperm tail, forms a complex with CFAP47 and participates in middle-piece flagellar assembly through IMT-IFT transport [129]. These studies provide further evidence for understanding the function of CFAP47 in sperm. ACTL7B protein is specifically localized to the developing acrosome, within the nucleus of early spermatids, and to the flagellum connecting region. It is necessary for maintaining the structural integrity of the sperm flagellar connecting piece, axoneme, and mitochondrial sheath, as well as ensuring the firm association of outer dense fibers, axonemal microtubules, and mitochondria [133].

## 4. Clinical Diagnosis and Treatment of MMAF Based on the Molecular Mechanisms

### 4.1. Diagnosis of MMAF

The diagnosis of MMAF requires the combined use of morphological analysis, genetic testing, and emerging technologies to ensure diagnostic accuracy and comprehensiveness. Wang et al. have systematically summarized the clinical examination methods for MMAF patients into three key components: routine clinical examination, ultrastructural examination, and genetic analysis [23]. In recent years, an increasing number of new technologies have been gradually introduced to help with the comprehensive and accurate analysis and diagnosis of MMAF. With transmission electron microscopy (TEM), ultrastructural evaluations can be performed to more precisely define the flagellar structural defects leading to MMAF, which also assists in determining the prognosis of these patients. However, due to the high costs, TEM is primarily used in the research setting. Whole-exome sequencing (WES) is a commonly used method for identifying unknown mutations in MMAF, and it is considered a suitable alternative to Sanger sequencing [136]. Additionally, new technologies provide more robust assistance in rapidly identifying fertility-related novel genes. Induction of STOP codons (iSTOP) is an efficient gene-editing tool, and a recent study employed iSTOP technology in founders’ germ cells, demonstrating that iSTOP could assist in directly modeling reproductive diseases and phenotypic analysis in the founders’ germ cells. This technique offers a time-saving approach to verifying genetic defects such as nonsense mutations [137]. Despite the benefits these new technologies offer for identifying MMAF, they still need to be closely integrated with traditional clinical examination methods.

### 4.2. Treatment of MMAF

Intracytoplasmic sperm injection (ICSI) is a method to help MMAF patients obtain their own genetic offspring. Favorable outcomes following ICSI treatment have been reported in several MMAF cohorts, with most couples obtaining healthy babies [138,139,140]. However, several reports also indicate failed ICSI treatments [141]. The varied prognosis following ICSI may be related to the different mutated genes causing MMAF, as mutations in different genes result in different structural damage that affects fertility. Patients with mutations in *DNAH1* [44,47,139], *CFAP43,* and *CFAP44* [140] generally achieve favorable outcomes with ICSI treatment, whereas *CEP135* is frequently mentioned in cases of ICSI treatment failure, which may be related to the crucial role that CEP135 protein plays in centrosome formation [8]. Beyond the sperm factors, ICSI treatment failure can also result from various other considerations, such as the female partner’s health status and issues related to fertilization and embryo development [114]. For example, Liu et al. reported that a patient with an *FSIP2* compound heterozygous mutation experienced failed ICSI treatment, whereas favorable results were reported for another MMAF patient with an *FSIP2* compound heterozygous mutation undergoing ICSI treatment [92,94]. Some studies suggest that the severity of the MMAF phenotype typically does not significantly affect ICSI outcomes [138]. The limited number of available MMAF patients places a constraint on concluding the efficacy of ICSI treatment, and these prognostic relationships should be confirmed in larger cohorts.

The majority of MMAF cases are autosomal recessive disorders. In recent years, alternative inheritance patterns, such as X-linked transmission, have also been reported [127]. Consequently, in cases where the partner is unaffected, most offspring of MMAF patients conceived through ICSI will be carriers of the mutation. However, if the mutation is located on the X chromosome, the offspring may either be affected by the disease or carry the mutation, depending on their sex. Furthermore, MMAF patients may have an increased risk of chromosomal abnormalities, leading to a higher likelihood of chromosomal defects and birth anomalies in children conceived via ICSI [142]. As such, the potential use of preimplantation genetic testing (PGT) to prevent the transmission of genetic risks to offspring is a matter that warrants careful consideration and debate [23]. In addition, genetic counseling plays a vital role in assessing and mitigating the risk of transmitting genetic defects through ICSI in these patients.

While ICSI has proven to be an effective method for overcoming the physical limitations of MMAF sperm, enabling some patients to obtain healthy offspring, it is not universally successful. Gene replacement therapy has been successfully developed to treat inherited retinal diseases [143]. Adoption of gene replacement therapy for MMAF needs to be handled with extra caution due to many unknown factors associated with genetic manipulation involving spermatogenesis [144].

## 5. Conclusions

With advances in diagnostic techniques, the number of cases of MMAF has seen a great rise in the last few years in diverse patient populations. The field has seen rapid progress as a number of functional studies have begun to shed light on the genetic pathogenesis of MMAF. Here, we have provided a systematic review of MMAF, particularly focusing on the molecular genetics of this disorder. In MMAF patients, the known pathogenic genes principally encode proteins essential for sperm flagellar assembly and motile functions, with a great number of proteins residing in the axoneme, peri-axonemal structures, and centrosome. With ever-increasing pathogenic genes and new mutations discovered, along with more effective study tools such as cryoelectron microscopy, a more complete understanding of the molecular pathogenesis of MMAF can be accomplished. Although ICSI treatment has offered hope for achieving fertility, more controlled studies are needed for improved outcome aided by a well structured genetic counseling program to provide more complete genetic and non-genetic information. 

## Figures and Tables

**Table 1:** The axonemal MMAF genes.

Gene Name	Location	Gene Variation	Human/KO Mice Phenotype	Specific Phenotype	Mechanism	Reference
*CFAP70*	Base of ODA	c.1723-1G>T	H ^1^	Typical MMAF phenotypes ^2^; the absence of the central pair and ODAs	Disorganization of ODAs and the central pair	[27]
		c.178T>A				
		c.2962C>T	H, M ^3^	Typical MMAF phenotypes; severe deformation of the axoneme; low ratio of axoneme assembly	Blocking the interaction among CFAP70, QRICH2 and TTLL5; the failure of cytoplasmic preassembly of CFAP61 and CFAP91	[28]
		-	M	Typical MMAF phenotypes; disorganized microtubules and ODFs	Hindering the assembly of ODAs via DNAI1 and DNAI2; blocking the IFT via AKAP3	[29]
*DNAH8*	Dynein γ-heavy chain of ODA	c.6158_6159insT	H	Typical MMAF phenotypes; disorganized or missing ODAs and microtubules	Disruption of the interaction between DNAH8 and DNAH17	[30]
		c.11771C>T plus c.6689A>G	H, M	Typical MMAF phenotypes; the lack of microtubules; misarranged or supernumerary ODFs; disassembled or absent ODAs; disorganized microtubules and ODFs in KO mice	Disruption of the interaction between DNAH8 and DNAH17	[31]
		c.9427C>T plus c.12721G>A				
		c.6962_6968del				
		c.378_379del	H	Typical MMAF phenotypes; missing or irregular arrangement of ODFs; the absence of the central pair; disorganized outer doublet microtubules	The absence of ODAs due to the disruption of the interaction between DNAH8 and DNAH17	[32]
*DNAH17*	Dynein β-heavy chain of ODA	c.1293_1294del	H	Typical MMAF phenotypes; abnormal MS; cytoplasmic bags containing unassembled flagellar components; the absence of the central pair and outer microtubule doublets	Damaging the ODA structure	[25]
		c.7994_8012del				
		c.5486G>A				
		c.10496C>T				
		c.10784T>C				
		c.10486_10497dup				
		c.4445C>T	H	Typical MMAF phenotypes; the absence of ODAs surrounded by a disorganized MS; defective FS	Damaging the ODA structure via the interaction with DNAI2	[33]
		c.6857C>T				
		c.5368C>T	H	Typical MMAF phenotypes; the absence of ODAs	Damaging the ODA structure	[34]
		c.13183C>T				
		c.6308C>T	H, M	Typical MMAF phenotypes; disorganizations of the FS and ODFs; KO mice showed disorganized flagellar cross-sections, disrupted MS and FS, and missing ODAs and microtubules	-	[6]
		c.11803C>T				
		c.5707C>T				
		c.12915+1C>T	H	Typical MMAF phenotypes; the absence of the central pair and outer doublet microtubules; disarranged ODFs	Impacting outer doublet microtubules 4–7 in sperm flagellar assembly	[35]
		c.13202G>A				
		c.8512-2A>G				
		c.13294C>T				
		c.12865_12867delTCC and c.13105C>T	H	Typical MMAF phenotypes	-	[36]
		c.612C>G and c.4237-6C>A				
		c.T2150C and c.G7136C				
		c.4810C>T	H	Typical MMAF phenotypes; thickened MS; disorganized ODFs; the absence of microtubules	Disruption of the interaction between DNAH8 and DNAH17	[37]
*CFAP43* and *CFAP44*	Next to the outer doublet microtubules 5–6 bridge and connected to the IDA	c.2802T>A and c.4132C>T (CFAP43)	H, M	Typical MMAF phenotypes; hypertrophy and hyperplasia of FS; the absence of central microtubules; distorted cytoskeletal components; KO mice showed a lack of the central pair and they had disordered outer doublet microtubules and ODFs	Impacting the intra-flagellar transport	[38]
		c.253C>T and c.3945_4431del (CFAP43)				
		c.386C>A and c.2802T>A (CFAP43)				
		c.2005_2006delAT (CFAP44)				
		c.3541−2A>C (CFAP43)	H, M	Typical MMAF phenotypes; defects in the ODFs, the FS, and the MS; partial or complete loss of the central doublet microtubules; disordered outer doublet microtubules; the worse motility in the *Cfap43*^−/−^ mice; the absence of the head of RS interacting with the central pair; substantial structural disorganization with uneven distribution of the nine outer doublet microtubules and the absence of the central pair complex in the *Cfap43*^−/−^ mice; defects in MS and FS in the *Cfap44*^−/−^ mice; the mislocalization of the central pair and the loss of ODF 3 and 8 in the *Cfap44*^−/−^ mice	Impairing the structure of the outer doublet microtubules 5–6; impacting the interaction between CFAP43 and CFAP44	[39]
		c.1240_1241delGT (CFAP43)				
		c.2658G>A (CFAP43)				
		c.2680C>T (CFAP43)				
		c.3882delA (CFAP43)				
		c.3352C>T (CFAP43)				
		c.1302dupT (CFAP43)				
		c.1040T>C (CFAP43)				
		c.2141+5G>A (CFAP43)				
		c.1890+1G>A (CFAP44)				
		c.3175C>T (CFAP44)				
		c.2818dupG (CFAP44)				
		c.1387G>T (CFAP44)				
		c.4767delT (CFAP44)				
		c.1140_1143del (CFAP43)	H	Typical MMAF phenotypes	-	[40]
		c.739A>T (CFAP43)				
		c.1474G>C (CFAP43)				
		c.4600C>G (CFAP43)				
		c.4963C>T (CFAP44)				
		c.2935_2944del (CFAP44)	H	Typical MMAF phenotypes	-	[41]
		c.T1769A (CFAP44)				
		c.G3262A and c.C1718A (CFAP44)				
		c.3661-2A>-(delA) (CFAP43)				
		c.585_735del (CFAP43)	H	Typical MMAF phenotypes; the loss of the central pair or severe disorganization of the FS, ODFs, and axonemal disassembly	-	[42]
		c.944del (CFAP43)				
		c.4579dup (CFAP43)				
		c.3768+1G>A (CFAP43)				
		c.386C>A (CFAP43)				
		c.1418C>T (CFAP43)				
		c.2546T>C (CFAP43)				
		-	M	Typical MMAF phenotypes; the manchette did not elongate and disengaged from the head to the tail; the direction of abnormal manchette microtubules was perpendicular to the assembly direction of the flagellum	Impacting the intra-manchette transport (IMT) associated with CFAP43	[43]
*DNAH1*	Dynein heavy chain of IDA	c.6912C>A, c.7076G>T et al.	H	A high percentage of short flagella and a low percentage of bent flagella	The absence of *DNAH1* removes the anchoring site of the RS3	[44]
		c.11726_11727del, c.4552C>T et al.	H	Typical MMAF phenotypes; the loss of sperm motility	-	[45]
		c.1336G>C and c.2912G>A	H	Typical MMAF phenotypes; the absence of the IDA and RS; the displaced dense fibers and microtubules	Impacting the expression of *DNALI1*	[46]
		c.1832T>C, c.2301-1G>T et al.	H	Typical MMAF phenotypes; the absence of the central pair; the absence of IDAs; the displacement of ODFs and/or the outer doublet microtubules; some unorganized axoneme clusters	Blocking the interaction between the C-terminus of DNAH1 in IDA3 and DNALI1; the absence of the RS3 anchoring site, resulting in the loss of the central pair	[47]
		c.8170C>T and c.4670C>T	H	Typical MMAF phenotypes; missing central pair and disordered outer doublet microtubule arrangements	-	[48]
		c.7435C>T	H	Typical MMAF phenotypes; the malformation or absence of the center pair; disorganized MS and FS	-	[49]
		c.10757T>C				
		c.11726_11727delCT				
		c.12154delC				
		c.10627-3C>G				
*DNAH2*	Dynein heavy chain of IDA	c.9298C>T	H	Typical MMAF phenotypes; missing central pair and disordered outer doublet microtubule arrangements; missing or fragmented ODFs; partitional or hypertrophic MS	Decreasing the expression of *DNALI1*	[4]
		c.5770C>T				
		c.11500C>T				
		c.6960C>A				
		c.11503T>C				
		c.A2116C	H	Typical MMAF phenotypes; absence of the central pair; disrupted IDA	Impairing the ATPase activity of DNAH2; decreasing DNAH1	[50]
		c.C11635T				
		c.A5507G				
		c.G9291T				
		c.G4774A				
		c.G5771C				
		c.12720G>T	H, M	Typical MMAF phenotypes; KO mice showed abnormally arranged MS, misaligned ODFs and microtubule doublets, disorganized RS and IDA	Impacting the interaction between DNAH2 and DNAH1; blocking DNAH2′s role in annulus migration; impacting the IFT	[51]
*DNAH6*	Dynein heavy chain of IDA	c.6582C>A	H	Typical MMAF phenotypes; the absence of the central pair; disorganization of the peripheral doublet microtubules and the ODFs; disorganized MS and FS	Reducing the ATPase activity and microtubule-binding capacity of DNAH6; destroying the flagellar axoneme assembly	[52]
		c.11258G>A				
		c.5264C>T and c.8726A>G	H	Typical MMAF phenotypes; the absence of the central pair; affected RS	Blocking the interaction among DNAH6, DNAH1 (IDA component), SPAG6 (central pair component), RSPH1 (RS component), AKAP4 (FS component), and ACTL7A (acrosome-associated protein)	[5]
		c.8852-1G>A and c.10127T>A				
		c.9250C>G				
*DNAH7*	Dynein heavy chain of IDA	c.2478dupA	H	Typical MMAF phenotypes; severe defects in the MS; severe IDA loss	Blocking the interaction among DNAH7, TBC1D21, and TOMM20	[53]
*DNAH10*	Dynein heavy chain of IDA	c.12838G>A	H, M	Typical MMAF phenotypes; the absence of IDAs; disorganization of axonemal or peri-axonemal structures; disorganized MS, ODFs, and microtubules in KO mice	Impacting the IMT and IFT	[54]
		c.7601C>T				
		c.5663G>A				
		c.11887C>T				
		c.7260dup				
		c.12235del				
		c.2514delG	H	Typical MMAF phenotypes; IDA deficiency	Impacting the interaction among DNAH10, DNAH1 (IDA component), DNAI1 (ODA component), SPAG6 (central pair component), and AKAP4 (FS component)	[55]
		c.10820T>C and c.12692C>T				
*DNHD1*	Dynein heavy-chain component	c.8782C>T	H	Typical MMAF phenotypes; the absence of the central pair complex; abnormal ODF and MS	-	[56]
		c.5989G>A				
		c.2581C>T and c.6031C>T				
*WDR63* (*DNAI3*)	Dynein intermediate chain of IDA	c.163C>T	H, M	Typical MMAF phenotypes; disorganized “9 + 2” axoneme combined with aberrant IDAs, ODAs, ODFs, FSs, and N-DRCs in KO mice	Impacting the IDA assembly by blocking the interaction among WDR63, WDR78, DNAH2, and DNAH10	[57]
		c.1075C>T				
*CCDC39* and *CCDC40*	Base plate component	c.983T>C (CCDC39)	H	Typical MMAF phenotypes; the absence of the central pair; the disorganized outer doublet microtubules	-	[58]
		c.901C>T (CCDC40)	H	Immotility; typical MMAF phenotypes	-	[59]
		c.2065_2068dup (CCDC40)				
		c.1072del and c.1007-1010del (CCDC39)	H	Typical MMAF phenotypes; unassembled axonemal and peri-axonemal components; severe tubular disorganization of the flagellar axoneme	The absence of CCDC39	[60]
		c.1675G>T (CCDC40)				
		c.248del and c.736_755dup (CCDC40)				
*CCDC65*	A component interconnecting microtubule doublets	c.1208del	H	Typical MMAF phenotypes; disorganization with abnormal doublet positioning; severe midpiece defects	Impairing the interaction among CCDC65, DNAI1 (located at the ODA), DNALI1 (located at the IDA), and SPAG6 (located at the central pair complex)	[61]
		c.1126C>T				
*DRC1*	Core structural component of the N-DRC	c.1296G>A	H	Typical MMAF phenotypes	-	[62]
		c.C1660T	H, M	Typical MMAF phenotypes; severe axonemal disorganization and unassembled microtubule doublets; KO mice showed the absence of sperm motility, separated ODFs, and the absence of N-DRCs, RSs, and dynein arms	Decreasing flagellum axoneme stability	[63]
		c.C238T				
*DRC4* (*GAS8*)	Core structural component of the N-DRC	c.1011+2T>C	H	Typical MMAF phenotypes; immotile sperm; defects in MS and FS; the absence of some peripheral microtubule doublets	Destroying the N-DRC structure; impairing the interaction among DRC4, DRC1, and CCDC65	[64]
*AK7*	Associated with the protein kinase A located at the RS	c.1846G>A	H	Typical MMAF phenotypes; low motility of the sperm	-	[65]
		c.1153A>T	H	Typical MMAF phenotypes; multiple axonemes in uniflagellate spermatozoa; mitochondrial vacuolization; the absence of a central pair	Impacts on the interaction between AK7 and DNAH protein family; destroying the regulatory function of AK7 on mitochondria	[66]
*CCDC189*	Attached to the RS	-	M	Impaired MS and coiled FS; structural abnormalities of microtubules	Blocking the interaction between CCDC189 and the RS via RSPH1 and CABCOCO1; impacts on the IFT via blocking the interaction with IFT20 and IFT88	[11]
*CFAP61*	Main component of the CSC	c.143+5G>A	H, M	Impaired motility of sperm along with typical MMAF phenotypes; KO mice showed separated microtubules and ODFs and lost certain RS complex components	Impacting the role of CFAP61 in the late stages of RS assembly	[67]
		c.451_452del	H	Typical MMAF phenotypes; disorganized axoneme structure along with the disappearance of microtubules, dynein arms, RS	Affecting the normal assembly of the central pair, RS, and IDA	[68]
		c.847C>T				
		c.1654C>T and c.2911G>A	H	Typical MMAF phenotypes; absence of central pair microtubules and MS malformation	-	[69]
		c.144-2A>G and c.1666G>A				
		c.1245+6T>C	H	Severe axoneme disorganization such as the absence of central or outer doublet microtubules, missing RS, and misshapen MS	Severe defects to CSC	[70]
*CFAP65* (*CCDC108*)	Unknown	-	M	Disorganized axoneme and peri-axoneme structures, such as absence or disorganization of outer doublet microtubules and ODFs; the absence of the central pair complex; the unstable formation of MS	Interfering with the interaction between CFAP65 and the RS component RSPH1	[71]
*CFAP91* (*MAATS1*)	A component of the CSC	c.682+1G>A	H	Typical MMAF phenotypes; the absence of the central pair complex; an abnormal number of ODFs	Destabilizing the RS and CSC structure; impacting the interaction among CFAP91, CFAP251, and radial spoke protein 3 (RSP3)	[72]
		c.124G>C				
*CFAP206*	Microtubule-docking adapter for the RS and IDA	c.1430dupA	H, M	Typical MMAF phenotypes; disorganized RS and CSC; distorted axoneme with a lack of peripheral doublets, the absence of the central pair, or abnormal distribution of ODFs in KO mice	Blocking the formation of RS and CSC	[73]
*CFAP251* (*WDR66*)	A component of the CSC	c.1192-3C>G	H	Higher rates of absent and coiled flagella; disorganization in axonemal and other peri-axonemal structures	-	[74]
*NPHP4*	A structural protein in the cilia	c.1490C>G	H	Typical MMAF phenotypes; “9 + 0” arrangements of microtubules with an absent central pair	Impairing of the interaction between NPHP4 and the central pair	[75]
*SPEF2*	A component of the central-pair-associated apparatus C1b	c.2507+5delG	H, M	Typical MMAF phenotypes; disorganized and scattered MS, ODFs, and FS; defects in both the central pairs and RS; unassembled axonemal components such as microtubule-like structure, fibrous elements, and mitochondria in KO mice	-	[76]
		c.C4096T				
		c.2649dupA				
		c.3400delA				
		c.3922dupA				

^1^ H refers to the MMAF phenotype in humans. ^2^ Typical MMAF phenotype refers to short, coiled, absent, and irregularly shaped flagella. ^3^ M refers to the MMAF phenotype in KO mice.

**Table 2:** The peri-axoneme-associated pathogenic genes.

Gene Name	Location	Gene Variation	Human/KO Mice Phenotype	Specific Phenotype	Mechanism	Reference
*ODF2*	A component of ODFs	c.202A>G	H ^1^	Tail deformity; high proportion of outer dense fiber deficiencies	Impairing the ODF assembly	[89]
*CFAP58*	The entire flagella and predominantly concentrate in the midpiece	c.2092C>T	H, M ^2^	Typical MMAF phenotypes ^3^; short and coiled flagella in KO mice	Causing defective flagellogenesis	[90]
		c.1429del and c.2092C>T				
		c.2052del				
		c.1696C>T				
		c.2274C>A				
		c.323C>T and c.1855C>T	H	Typical MMAF phenotypes		[91]
		c.1883A>G and c.2020G>T				
*FSIP2*	A component of FS	c.16246_16247ins CCCAAATATCACC and c.17323C>T	H	Typical MMAF phenotypes	Disrupting sperm flagellar development	[92]
		c.8368_8369insC	H, M	Typical MMAF phenotypes; dysplastic and disorganized MS; absent FS; MMAF phenotypes, absent FS, and exposed axonemes in knock-in mice		[93]
		c.1494C > A and c.11020_11024del	H	Typical MMAF phenotypes; abnormal mitochondrial arrangement and disorganization; dysplastic FS		[94]
		c.1750T>A and c.13600A>G	H	Typical MMAF phenotypes; abnormal heads		[95]
*AKAP3*	A structural protein of FS	c.2286_2287del	H	Typical MMAF phenotypes; abnormal acrosomal morphology	Affecting FS assembly	[96]
		c.44G>A				
*AKAP4*	A structural protein of FS	c.1285C>T	H	Typical MMAF phenotypes; missing or inhomogeneous FS	Affecting the interaction with QRICH2 and its normal expression	[97]

^1^ H refers to the MMAF phenotype in humans. ^2^ M refers to the MMAF phenotype in KO mice. ^3^ Typical MMAF phenotype refers to short, coiled, absent, and irregularly shaped flagella.

**Table 3:** The centrosome-associated pathogenic genes.

Gene Name	Location	Gene Variation	Human/KO Mice Phenotype	Specific Phenotype	Mechanism	Reference
*CEP135*	Centriole	c.A1364T	H ^1^	Typical MMAF phenotypes ^2^	Massive formation of filamentous aggregates impairing centriole biogenesis	[8]
*DZIP1*	Centrioles and pericentriolar matrixes	c.188G>A	H, M ^3^	More than 90% have no flagellate; absent or very short axoneme; absent flagella, cytoplasm residual, and abnormal heads in KO mice	Centrosome damage caused by the absence of DZIP1	[7]
		c.690T>G				
*PRSS50*	Murine sperm midpiece	-	M	-	PRSS50-NF-κB-LRWD1 pathway affecting the level of LRWD1 and AKAP4	[108]
*CEP78*	Distal region of mature centrioles	c.1629-2A>G	H, M	MMAF phenotype; head abnormalities; multiple abnormalities of sperm head and flagella in KO mice; defective microtubule arrangements and elongated centrioles in KO mice	Affecting the interaction and stability with IFT20 and TTC21A, resulting in an influence on the regulation of centrosomes and ciliary length	[109]

^1^ H refers to the MMAF phenotype in humans. ^2^ Typical MMAF phenotype refers to short, coiled, absent, and irregularly shaped flagella. ^3^ M refers to the MMAF phenotype in KO mice.

**Table 4:** The pathogenic genes related to the flagellar assembly.

Gene Name	Location	Gene Variation	Human/KO Mice Phenotype	Specific Phenotype	Mechanism	Reference
*TTC21A* (*IFT139A*)	An IFT complex component	c.3450+2delT	H ^1^	Typical MMAF phenotypes ^2^; absent or misplaced central-pair microtubules and peripheral doublet microtubules	-	[9]
*TTC29*	A potential member of the IFT-B complex	c.254+1G>A and c.1185C>G	H	Typical MMAF phenotype; abnormally shaped heads; defective sperm acrosomes	Affecting the development of flagella	[114]
*WDR19*	A core component in IFT-A	c.3811A>G	H	Typical MMAF phenotype	Lead to the abnormal expression of other IFT partials	[115]
*IFT74*	A core component in IFT	c.256G>A	H	Typical MMAF phenotype; abnormal mitochondrial sheath; cytoplasmic bags containing unassembled flagellar components; missing some peripheral doublets	Affecting IFT74 mRNA splicing and inducing mutant proteins with abnormal subcellular localization along the flagellum	[116]
*CCDC38*	Manchette and sperm tail	-	M ^3^	MMAF phenotype in KO mice	Affecting ODF2 transportation	[117]
*CCDC146*	Sperm connecting piece and sperm tail	-	M	MMAF phenotype; abnormal sperm heads in KO mice	Affecting interaction with CCDC38 and CCDC42 in the IFT pathway	[118]
*ARMC2*	Axonemal central pair complex	c.182C>G	H	Typical MMAF phenotype; complete absence of central pair complex; axonemal ultrastructure disorganization	-	[119]
		c.1264C>T	H	Typical MMAF phenotype; missing central microtubule pairs	Affecting flagellar structures	[120]
		c.314C>T	H	Typical MMAF phenotype; abnormal expression and localization of TOMM20 and AKAP4	Affecting CPC assembly/stability and flagellar assembly	[121]
		c.2227A>G				

^1^ H refers to the MMAF phenotype in humans. ^2^ Typical MMAF phenotype refers to short, coiled, absent, and irregularly shaped flagella. ^3^ M refers to the MMAF phenotype in KO mice.

**Table 5:** Other pathogenic genes.

Gene Name	Location	Gene Variation	Human/KO Mice Phenotype	Specific Phenotype	Mechanism	Reference
*CFAP47*	-	c.7154T>A	H ^1^, M ^2^	Typical MMAF phenotype ^3^; bent flagella in KO mice	-	[127]
		c.5224A>G				
		c.8668C>A				
	-	c.1414G>A	H	Typical MMAF phenotype; numerous malformed sperm heads, defective sperm annulus, and aplasia sperm mitochondrial sheaths	Influencing the expression of CFAP65, CFAP69, and SEPTIN4 to regulate spermatogenesis	[128]
	-	c.706G>A and c.1337C>T	H	Typical MMAF phenotype	Forming a complex with WDR87 and participating in the assembly of the midpiece of flagella via IMT-IFT transport	[129]
*STK33*	-	c.1235del	H	Typical MMAF phenotype	-	[130]
*CFAP74*	-	c.983G>A and c.3532G>A	H	Lost or short tail defects and an incomplete mitochondrial sheath	Affect the ultrastructure of the sperm axoneme and MSs	[131]
		c.652C>T and c.4331G>C				
*BRWD1*	-	c.C523T	H	Typical MMAF phenotype; missing ODAs and IDAs	-	[132]
		c.A5573T				
		c.G166A and c.T1016C				
*ACTL7B*	Developing acrosome; within the nucleus of early spermatids; flagellum connecting region	-	M	Conspicuous absence of the flagella connecting pieces; severe disruptions in flagellar midpiece structure in KO mice	Affecting the structural integrity of the sperm flagellar junction, axial filament, and mitochondrial sheath	[133]

^1^ H refers to the MMAF phenotype in humans. ^2^ M refers to the MMAF phenotype in KO mice. ^3^ Typical MMAF phenotype refers to short, coiled, absent, and irregularly shaped flagella.
